# Recent Advances in Self-Powered Electrochemical Systems

**DOI:** 10.34133/2021/4673028

**Published:** 2021-03-12

**Authors:** Linglin Zhou, Di Liu, Li Liu, Lixia He, Xia Cao, Jie Wang, Zhong Lin Wang

**Affiliations:** ^1^Beijing Institute of Nanoenergy and Nanosystems, Chinese Academy of Sciences, Beijing 100083, China; ^2^College of Nanoscience and Technology, University of Chinese Academy of Sciences, Beijing 100049, China; ^3^School of Materials Science and Engineering, Georgia Institute of Technology, Atlanta, GA 30332, USA

## Abstract

Electrochemistry, one of the most important research and production technology, has been widely applicated in various fields. However, the requirement of external power source is a major challenge to its development. To solve this issue, developing self-powered electrochemical system (SPES) that can work by collecting energy from the environment is highly desired. The invention of triboelectric nanogenerator (TENG), which can transform mechanical energy into electricity, is a promising approach to build SPES by integrating with electrochemistry. In this view, the latest representative achievements of SPES based on TENG are comprehensively reviewed. By harvesting various mechanical energy, five SPESs are built, including electrochemical pollutants treatment, electrochemical synthesis, electrochemical sensor, electrochromic reaction, and anticorrosion system, according to the application domain. Additionally, the perspective for promoting the development of SPES is discussed.

## 1. Introduction

Electrochemistry, which refers to the interrelation of electrical and chemical effects, has play a crucial part in the sustainable advancement and innovation of industrial processes, including chemical industry, medicine, materials, energy, metal corrosion, and environmental science [1–5]. Electrochemical process highly depends on external power supply, which aggravates the crisis of energy shortage and environmental pollution problems in modern society. To solve these issues, developing a self-powered electrochemical system (SPES), which can operate by integrating electrochemical system with energy harvesting technology for collecting energy form environment [6–10], is one of the promising approaches. On account of the sufficient availability of mechanical energy, converting it from environment to electricity has aroused broad attentions. Recently, many technologies that extract mechanical energy have been reported, such as electromagnetic generator [11] and piezoelectric nanogenerator [6]. However, the low energy conversion efficiency of electromagnetic generator at low frequency and low output power of piezoelectric nanogenerator limit their practical application.

Based on the conjunction of triboelectrification and electrostatic induction, triboelectric nanogenerator (TENG), also known as *Wang generators*, was invented to extract energy from a variety of ambient mechanical motions, like sound, water waves, and mechanical vibration [12–15]. TENG exhibits many unique advantages including light structural simplicity, diverse materials options, and high conversion efficiency [16]. Moreover, TENG has demonstrates its potential applications in micro/nano power sources [17, 18], self-powered (SP) sensors [19, 20], large-scale blue energy [21, 22], and direct high voltage power sources [22, 23]. To promoting the practical application of TENG, large researches have been focused on improving its output performance via investigating the basic principles [24], enhancing the surface charge density [25–30], and power management [31–33]; thus, 500 W·m^−2^ of area power density and >50% of conversion efficiency have been achieved, respectively [34]. In view of these advantages, TENG can be served as a promising alternatively energy harvesting power source to integrate with electrochemistry for electrochemical operation. Currently, many SPESs based on TENG have been developed, which will be elaborated in further detail below. Compared with traditional electrochemical systems, SPESs can drive the electrochemical process without the external power supply, which largely promote the sustainable development of electrochemical systems.

**Figure 1:**
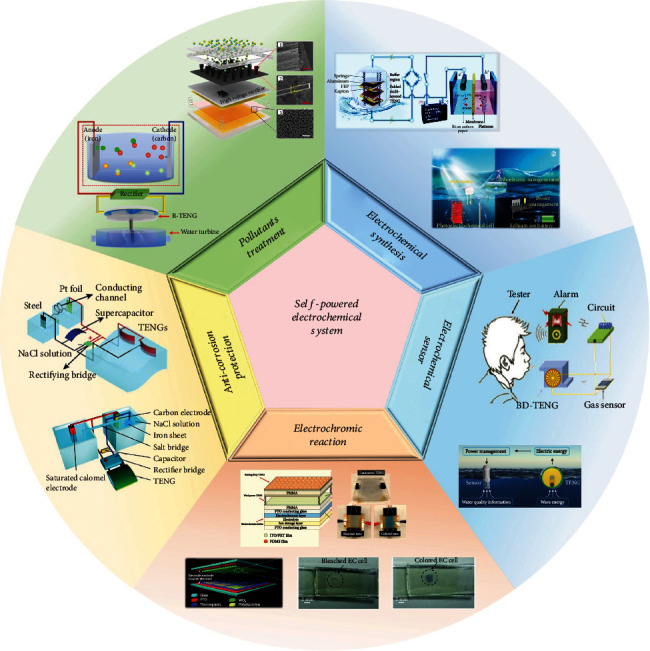
Diverse applications of self-powered electrochemical system. SP electrochemical system for pollutants treatment [50, 63], electrochemical synthesis [70, 78], electrochemical sensor [89, 95], electrochromic reaction [99, 100], and anticorrosion protection [112, 113].

Here, the recent progresses and practical applications of SPES based on TENGs are summarized. According to the application, the SPES is classified into five major applications including pollutants treatment, electrochemical synthesis, SP sensors, electrochromic reaction, and anticorrosion system (Figure 1), respectively. Additionally, perspectives and challenges for promoting the development of SPES are proposed. We would very much like that this paper will significantly advance the development of TENG in the electrochemistry field and offer a direction for future research in SPES.

## 2. Self-Powered Electrochemical System

SPES is developed from the combination of electrochemistry and TENG technology, where electrochemical process can operate by TENG that produces electrical power from the ambient environment. As a mechanical energy harvester, the recent development of TENG has been reviewed from the mechanism to the potential applications [35, 36]. Classified according to the motion direction and the capacitance change, four basic working modes of TENG were proposed since its first invention in 2012 [37–41], which can be divided into two types including contact-separation-type and sliding-type TENG. Driven by external force, repetitive changing of the distance between two planes and the size of contacting area will cause the variation of capacitance of TENG, resulting in the potential difference in two electrodes. For balancing the potential difference, electrons will flow back and forth in external circuit and thus generates an alternative current output. For driving the electrochemical process, a direct current is always employed in the current researches about SPES, where the alternative current output of TENG should be firstly transformed to direct current output by using rectifying device and then drive the electrochemical process.

The fundamental physics mechanism of TENG derives from Maxwell's displacement current [42, 43], which is defined as

(1) $$\begin{array}{c} J_{D}=\frac{\partial D}{\partial t}=\varepsilon \frac{\partial E}{\partial t}+\frac{{\partial P}_{s}}{\partial t}, \end{array}$$

where *J*_*D*_, *D*, *P*_*S*_, and *E* are the total displacement current density, the electric displacement vector, the polarization field, and the electric field, respectively. The displacement current (*I*_*D*_) is a surface integral of *J*_*D*_:

(2) $$\begin{array}{c} I_{D}=\int J_{D}dS=\int \frac{\partial D}{\partial t}dS=\frac{\partial }{\partial t}\int \nabla ∙Ddr=\frac{\partial }{\partial t}\int \rho dr=\frac{\partial Q}{\partial t}a, \end{array}$$

where *S*, *ρ*, and *Q* are the medium surface, the distribution of free charges, and the total free charges on the electrode. According to the equation, the output loop of TENG contains two portions, including the internal circuit in TENG that is controlled by displacement current and the observed current in the external circuit. In summary, the core of physics for current production is the internal driving force of *∂ ***P**_*s*_/*∂t*, which is named as the *Wang term* in the displacement current [43], and the external manifestation of displacement is the observed capacitive conduction current in external circuit.

## 3. Applications of Self-Powered Electrochemical System

### 3.1. Pollutant Treatment

Owing to the modern industrial activities, an alarming increase of toxic pollutants in the environment caused by human activities has brought to serious environmental issue, such as water pollution and air pollution, which is the most important environmental factor of disease and premature death in the world today [44]. For the growing threat of environmental pollutants, high efficiency pollutant treatment strategy is highly desired to ensure clean environment and human health. Due to high removal efficiency, great versatility, high amenability, and excellent environmental compatibility, electrochemical technology has been extensively developed as a promising method for environmental pollutants treatment [45, 46]. However, the requirement of an external power supply has become the major challenge for the practical application of electrochemical technology. Due to the invention of TENG by collecting mechanical energy from environment, SPES has been proposed as a candidate for environment treatment [47]. Furthermore, with the gradually enhanced output performance of TENG, many efforts have been devoted for removing environmental pollutants from water and air.

**Figure 2:**
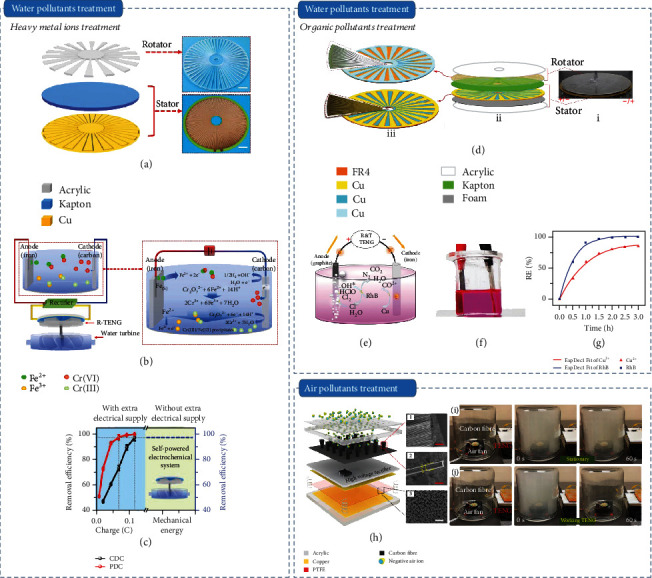
Self-powered electrochemical pollutant treatment: (a) sketch map of rotary TENG; insert displays the pictures of stator and rotator; (b) SP system for removing Cr(VI) powered by rectified TENG; (c) the comparison of charge consumption for Cr(VI) removal under different conditions [50]; (d) sketch map of the rotary TENG; (e, f) the mechanism image and optical image of SP electrochemical system for cupric ions and rhodamine B removal; (g) removal efficiency of cupric ions and rhodamine B [54]; (h) sketch map of the triboelectric negative air ion generator; (i, j) the contrast photographs of the smog cleaning by the MSNG in stationary conditions (i) and with the FS-TENG conducting at 0.25 Hz (j) [63].

Water pollutants are mainly composed of inorganic, organic, and biological contaminants. As one of the most toxic pollutants, heavy metal ions can biologically accumulate upon cumulative exposure, which is not biodegradable, and thus threaten human health via the entire food chain [48]. Aiming to remove heavy metal ions from environment, Li et al. reported a water-driven TENG to extract the kinetic energy from wastewater flow, which used to drive electrochemical reaction for Pb^2+^ and Cu^2+^ removal [49]. By using the integrated SP system, 97.4% of the two metal ions was removed from the wastewater in 100 min. Through comparing the electrochemical property of Cr(VI) driven by continuous DC (CDC) and pulsed DC (PDC), Zhou et al. confirmed an enhanced efficiency of removing Cr(VI) under PDC than that of CDC due to the better utilization of ferrous ion, the lower electrode passivation, and the higher ion diffusion rate during the reaction process [50]. Therefore, they proposed an SPES based on TENG with PDC output for improving the electrochemical performance of heavy metal ion treatment. The structures of the rotary TENG and SPES are shown in Figures 2(a) and 2(b). The charge consumption under different power supply is shown in Figure 2(c). Under equal charges consuming with 0.048 C, the removal efficiency driven by PDC was increased by 53.5% compared to that of CDC.

Organic pollutants in water have also drawn wide concern because of the most toxic and potentially carcinogenic; therefore, large efforts have been devoted to removing organic pollutants from wastewater. SP electrooxidation is the common process for organic pollutants treatment by using the produced chlorine and hypochlorite. Li et al. proposed a unique SP electrooxidation system to phenol removal via creatively employing *β*-cyclodextrin to increase triboelectrification [51]. In the condition of wastewater wave with velocity of 1.4 m s^−1^ and initial concentration of phenol with 80 mg L^−1^, 90% of the phenol was removed by the generated power in 320 min. Gao et al. reported a free-standing mode TENG integrated with electrocatalytic technology for degrading 4-aminoazobenzene [52]. With a commercial aluminum panel as stator, the power density reached up to 2.28 W m^−2^. Driven by the TENG, 4-aminoazobenzene can be electrochemical degradation to small molecule polymers by sensibly adjusting the potentials of electrochemical oxidation. By using the sponge to improve the contact intimacy and precharge injection to increase the surface charges in the dielectric film, Gao et al. improved the power density of a multilayer linkage TENG to 7.4 W m^−2^, which was used to drive electrochemical catalysis for degrading methyl red [53]. Powered by the multilayer linkage TENG, the degradation percentage of methyl red was almost 100% after 160 min, with a color change from red to colorless. Additionally, Chen et al. fabricated an SP multifunctional system which can simultaneously realize heavy metal ions and organic pollutant removal driven by a rotary TENG [54]. The illustrations of the rotary TENG and system are shown in Figures 2(d) and 2(e). After rectifying, 100% of rhodamine B and 97.3% cupric ion were removed after 3 hours treatment (Figures 2(f) and 2(g)). Besides, Yang et al. reported an SP electrocatalytic system containing a hybrid energy cell including TENG to degrade methyl orange, which can further achieve a higher performance [55]. In this system, the generated energy can directly power the electro-degradation of methyl orange or store in an energy storage unit before utilizing for methyl orange treatment, where the removal efficiency of methyl orange reached 80% after 144 h.

Owing to merits of environmentally friendly and particularly efficient, SP electrochemical advanced oxidation system such as SP electro-Fenton process is proposed to removing organic pollutants from the water [56–58]. Feng et al. integrated a rotary TENG with an electrochemical cell to build an SP electro-Fenton system to remove dyes [59]. In this work, a modified graphite felt cathode was used to produce H_2_O_2_ along with •OH, and a platinum sheet anode was applied to generate active chlorine, which can be used to oxidize organic pollutants. Through power management, the electric power of integrated rotary TENG improved by 3.5 times for driving electrochemical reaction. Driving by wind flow at the speed of 6.2 m s^−1^, the SP electro-Fenton system can efficiently degrade dyes without aerating oxygen, and a removal efficiency of 87.5% within 120 min was achieved. Combining with 3D printing techniques, Tian et al. prepared a 3D printed elastic TENG to form an SP electro-Fenton system for removing methylene blue, where 97.0% of methylene blue was removed within 140 min [60].

Biological contaminants such as harmful bacteria and algal blooms caused by uncontrolled discharge of wastewater are the other kinds of water pollutants. Jiang et al. reported an SP electrochemical water treatment system on the basis of an arch-shaped TENG for cleaning sterilization and algae in wastewater [61]. By extracting water wave energy, most of the bactericidals and algae treatment were cleaned by the produced Cl_2_ and reduced graphene oxide. By using SPES, high removal efficiencies for three model bacteria and mixed marine algae were achieved.

Air pollution including fine air particulate matter (PM) and gaseous pollutants is a crucial toxicant caused by human activities. Generally, air pollution exposure is related to lots of chronic diseases, such as pulmonary and cardiovascular disease [62]. To clean PM 2.5, Guo et al. reported an SP triboelectric negative air ion generator (MSNG) powered by TENG [63]. Powering by TENG, the voltage of carbon fiber electrodes was over 2 000 V, which can be employed to produce negative air ions in these electrodes (Figure 2h). For demonstrating the ability to clean PM 2.5, 1 × 10^13^ NAIs was generated by using a palm-sized MSNG, and PM 2.5 with initial concentration of 999 *μ*g m^−3^ was quickly declined to 0 *μ*g m^−3^ in 80 s at 0.25 Hz. Furthermore, the observable heavy smog purification processes are displayed in Figures 2(i) and 2(j), which demonstrated the high efficiency of the MSNG for air purge.

The primary pollutants of gaseous pollutants result from the original pollutants directly discharged into the atmosphere from the source, mainly including oxysulfide, oxynitride, and organic compounds [64]. For removing sulfur dioxide (SO_2_) and dust, Chen et al. reported an SP air cleaning system on the basis of a rotary TENG [65]. Driven by nature wind, rotary TENG produced a high voltage about 300 V, which was applied to power the electrochemical oxidation of SO_2_ without the byproducts such as ozone and NOx than conventional electrostatic precipitation. For cleaning oxynitride, Han et al. proposed an SP NO_X_ absorption and degradation system on the basis of a radial-engine-shaped TENG system [66]. By harvesting win energy, the SP system synchronously achieved the removal of NO_X_. For cleaning formaldehyde in indoor atmosphere, Feng et al. demonstrated an SP electrostatic filter by integrating TENG with photocatalysis technology [67]. In this work, a single electrode TENG was used as power source to produce high electric field on the filtering network. Consequently, the SP electrostatic filter was demonstrated to clean formaldehyde via both electrostatic adsorption effect induced by TENG and enhanced photocatalytic effect by the photocatalyst on the SP filter networks, where the formaldehyde concentration decreased to 60% within 250 min, and a tripled increased efficiency of formaldehyde removal was achieved.

### 3.2. Self-Powered Electrochemical Synthesis System

**Figure 3:**
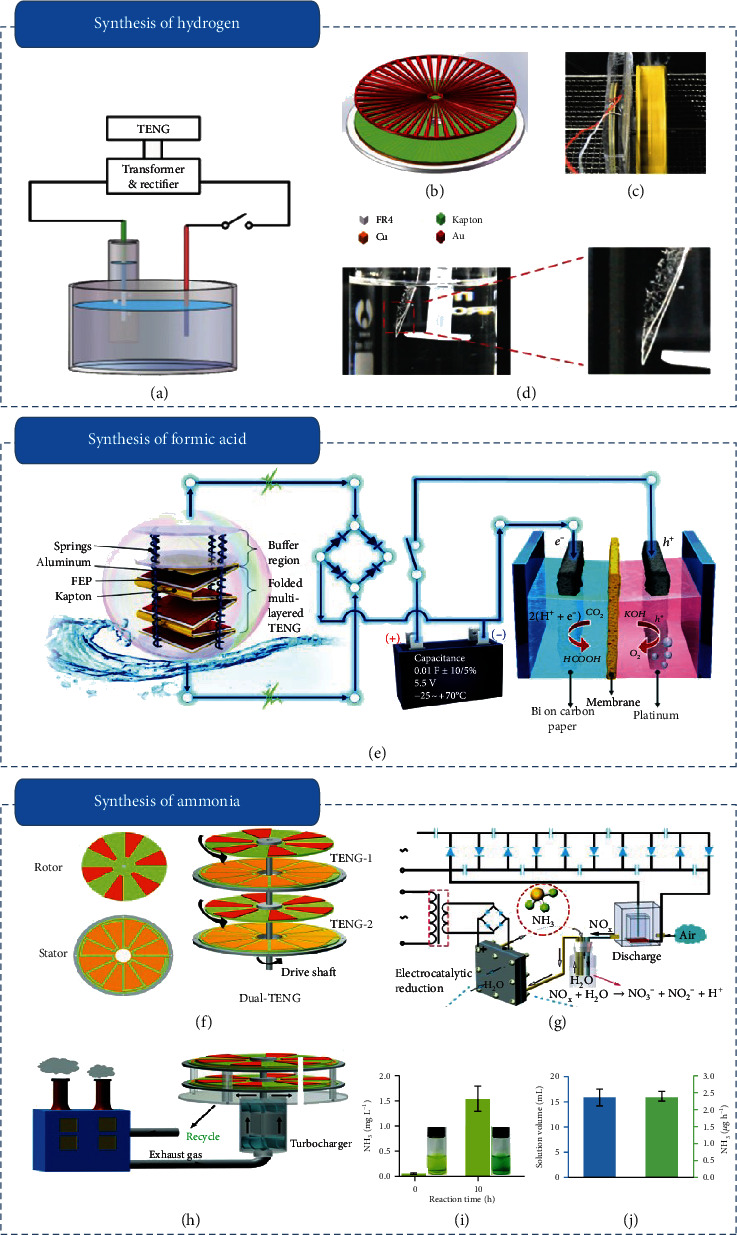
Self-powered electrochemical synthesis: (a) sketch map of the SP water splitting system; (b) structure of the disk TENG; (c) the SP water splitting system driven by a miniature water turbine; (d) images of the system with bubble producing [74]; (e) sketch map of the SP electrochemical system for CO_2_ reduction powered by TENG for harvesting ocean-wave energy [70]; (f) sketch map of the designed TENG; (g) reaction of SP ammonia synthesis system; (h) sketch map of the SP structure driven by exhaust gas; (i, j) concentration of NH_3_ in the cathode compartment at different times (i) and the volume of residual solution and the yield of NH_3_ (j) by the SP system after ten hours [82].

Converting energy from environment into easily storable chemical energy such as hydrogen and formic acid has drawn public attention as the alternative technology to ensure a clean and sustainable energy supply [68]. Generally, the process of clean fuel production needs an external input power, where renewable energies such as solar, wind, geothermal, and hydro have been applied along with carbon dioxide and water to generate clean fuels [69]. Owing to the ability of harvesting ambient mechanical energy, TENG-based SP electrochemical system has been demonstrated as a promising technology for clean fuel generation [70–73]. Tang et al. firstly proposed a fully SP water splitting system for producing hydrogen [74], which integrated a rotary TENG with a water splitting unit (Figures 3(a) and 3(b)). Driven by the rotary TENG at 10 Hz, the production ratio of hydrogen was up to 6.25 × 10^−3^ mL min^−1^ in 30% (w.t.) potassium hydroxide solution. As demonstrating, obvious bubbles were observed in the electrode (Figures 3(c) and 3(d)). To improve the production speed of hydrogen, introducing a hybrid cell to increase the output power of SP water splitting system is a promising strategy [75]. Yang et al. designed an SP water splitting system on the basis of a hybrid energy cell, where the hydrogen production speed had been improved to 4 × 10^−4^ mL s^−1^ [76].

As the other type of clean fuel, formic acid with characteristics of high volumetric capacity, low toxicity, and flammability under ambient condition has drawn wide attention [77]. Leung et al. presented an SP carbon dioxide reduction system that harvests energy from ocean wave for converting carbon dioxide into formic acid (Figure 3(e)) [78]. At an optimal value of discharge voltage (2.4 V) for each cycle, the system realized near 100% faradaic efficiency for shifting carbon dioxide into formic acid. Driven by TENG under a simulated waves with water surface area of 0.04 m^2^, the system generated 2.798 *μ*M of formic acid per day.

Ammonia (NH_3_) acts a crucial role in food production, industrial manufacturing, and a predictable ideal energy carrier in the future [79]. In industry, Haber-Bosch process is commonly employed to produce NH_3_ with the existence of hydrogen and external energy supply, where the challenge of grueling conditions increases the cost of production [80]. Due to a controllable operation under mild conditions utilizing mechanical energy by TENG, the SP electrocatalytic NH_3_ synthesis system based on TENG provides a promising candidate for the conversion of N_2_ to NH_3_. Gao et al. reported an SP sustainable metal-free NH_3_ production system based on a multilayer asway TENG by 3D printing technology, which can efficiency convert N_2_ into NH_3_ [81]. The maximum power density of 6.7 W m^−2^ was realized by the printed TENG, which was used to drive the production of NH_3_. By using the carbon materials from melamine sponge as the metal-free electrocatalyst, the assembled SP NH_3_ production system can reach NH_3_ yield of 36.41 *μ*g h^−1^ mg^−1^_cat._. By introducing a high-output dual-TENG configuration, Han et al. constructed an SP electrocatalytic NH_3_ synthesis system to simultaneously achieve nitrogen fixation and electrocatalytic reduction with air as the N_2_ source [82]. The electrocatalytic NH_3_ synthesis system mainly consisted of three parts, including TENG-1, a needle-plate, and TENG-2. Specifically, TENG-1 was used to produce high voltage, a needle-plate was utilized to fix nitrogen and produce NO_X_ which then flowed into a water receptacle to produce NO_3_^−^ and NO_2_^−^, and TENG-2 was employed to generate pulsed DC which drove the reaction for ammonia production (Figures 3(f) and 3(g)). Driven by the gas flow, a high voltage of 7 kV supplied by TENG-1 and the output performance of 3.1 V and 8.9 mA for the cell reaction supplied by TENG-2 were achieved, respectively (Figure 3h). By employing the SP electrocatalytic NH_3_ synthesis system based on above dual-TENG, 2.4 *μ*g h^−1^ of NH_3_ was successfully synthesized (Figure 3(i) and 3(j)).

Besides the abovementioned works, some other SPESs such as SP electrochemical oxidation system and SP electrodeposition system were also developed to electrochemical synthesis [83–86]. Zheng et al. reported an SP electrochemical oxidation system on the basis of designed cross-linked TENG for synthesizing polyaniline [85]. As for the cross-linked TENG, the high capability of 69.9 *μ*A and 845.6 V was achieved. By harvesting vibration energy, the SP electrochemical oxidation system was utilized as electricity source for converting aromatic amines to polyaniline. Wang et al. established a self-powered electrodeposition system for polypyrrole synthesis, where the polypyrrole as the electrode material of TENG was produced by TENG [86].

### 3.3. Self-Powered Electrochemical Sensor System

Based on TENG, lots of SP electrochemical sensors have been proposed to detect chemical substances. According to the mechanism of sensing, SP electrochemical sensor can be classified into two types including SP electrochemical passive sensor and SP electrochemical active sensor [47, 87].

SP electrochemical passive sensor is that the conventional sensors are driven by TENG for collecting mechanical energy from environment. Zhang et al. proposed an SP glucose biosensor based on contact-separation type TENG combined with lithium-ion battery [88]. The flexible TENG fabricated by a patterned polydimethylsiloxane (PDMS) film can extract energy from the motion of clapping; thus, the battery with an increased charging voltage from 400 mV to 800 mV was achieved after more than two hours, which was successfully demonstrated to power a glucose biosensor. Aiming to voluntarily supervise the water quality, Bai et al. reported an in situ SP sensing system that can convert the water wave energy to electricity based on tandem disk TENG [89]. Owing to the radial grating disk structure with swinging mass blocks, the tandem disk TENG driven by water waves can realize a conversion from low-frequency water wave motions into high-frequency output; thus, an average power density of 7.3 W m^−3^ was achieved. Through rectification and energy storage, the electricity produced by TENG can be utilized to drive electronics for monitoring the water quality.

**Figure 4:**
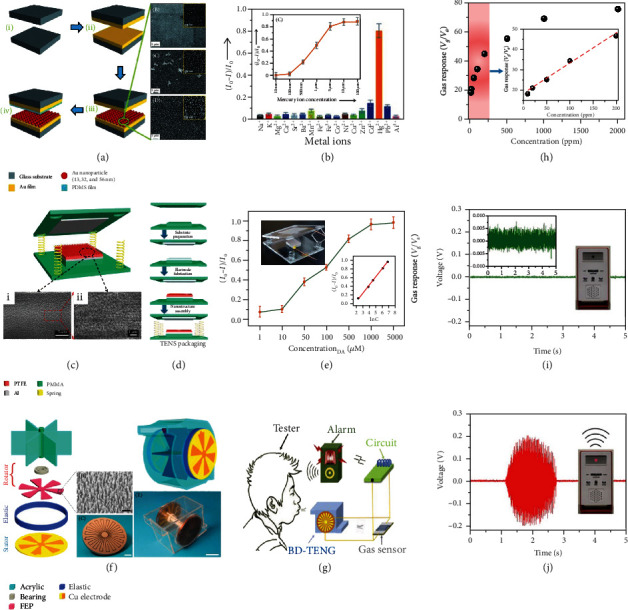
Self-powered electrochemical sensor: (a) fabrication process and the structure of the TENG; (b) selectivity of the SP electrochemical sensor for testing Hg^2+^ ions. Inset shows the sensitivity of Hg^2+^ ions detection [93]. (c, d) Sketch map and the fabrication process of the TENG; (e) sensitivity of the SP active sensor in supervising dopamine. Insets show the structure of designed TENG and proportional relation between output performance of TENG and the concentration of dopamine [94]. (f) Sketch map of the blow-driven TENG; (g) a schematic illustration the blow-driven TENG using as an SP breath analyzer; (h) response curve of the measured gas in terms of output voltage. Inset is the schematic illustration of a self-power breath analyzer; (i, j) voltage signals of the system with warning alarm by a person before and after drinking alcohol [95].

As for the SP electrochemical active sensor, a TENG is designed to actively generate electrical signal for responding the stimulation of chemical molecules or environmental factors such as ethanol, phenol, catechin, and pH, where the output performance of TENG and the target shows a linear relationship [51, 90–92]. Zhang et al. demonstrated SP sensors based on the TENGs fabricated by polyamide (PA) film and polytetrafluoroethylene (PTFE) film for detecting liquid/gaseous ethanol [90]. The TENG was mainly composed of two plates, where a layer of PA or PTFE film was pasted on a copper foil as back electrode, and a nanopore modified Al foil was used as the triboelectric electrode. Due to the degrees of wettability of the PA and PTFE films to ethanol, the output voltage of the SP sensor logarithmically declined with the concentration of ethanol solutions increasing from 20% to 80%, as well as the output signal decreased with the increase of ethanol gas concentration ranging from 40% to 80%. Lin et al. proposed a contact-separation-type TENG as an SP nanosensor toward catechin detection [91]. For this TENG, PTFE film and a layer of TiO_2_ nanomaterial (nanowire and nanosheet) array were used as a pair of triboelectric materials. Because of the strong interaction between Ti atoms of TiO_2_ nanomaterial and enediol group of catechin, a high sensitivity (detection limit of 5 *μ*M) and a linear range from 10 M to 0.5 mM of the SP nanosensor were achieved, demonstrating great potential for the determination of catechin concentrations in real samples. Wu et al. reported an SP triboelectric sensor based on the sliding type TENG for detecting pH value from a periodic contact/separation motion [92]. A fork-finger structure was designed for SP triboelectric sensor, which mainly consisted of fluorinated ethylene-propylene (FEP) film and metal electrodes. The reciprocating motion between SP triboelectric sensor and buffer solution resulted in charge transfer between the adjacent Cu bottom electrodes, generating AC voltage in the external circuit. The output voltage of the SP triboelectric sensor enhanced with increased pH value due to the increased ion concentration. Therefore, the pH value of buffer solution can be actively monitored in real-time by reading the output voltage. Li et al. proposed an SP active sensor to Hg^2+^ ions monitoring based on TENG where 3-mercaptopropionic acid-modified gold nanoparticles was used as recognition element [93]. In this system, a contact-separation-type TENG was employed, which shown a layered structure based on two plates (Figure 4(a)). The output performance of TENG and the concentration of Hg^2+^ ions displayed a linear relationship because the chemical potential difference between the different triboelectric layers defined the triboelectrification effects. As shown in the inset of Figure 4(b), the output performance of the TENG declined with improving the concentration of Hg^2+^ ions, where the short-circuit current ratio was proportional to the concentration of Hg^2+^ ions in the range of 100 nM-5 000 nM. Due to the high selectivity of 3-mercaptopropionic acid toward Hg^2+^ ions, Hg^2+^ ion can be specifically detected by the proposed sensing system (Figure 4(b)). Jie et al. demonstrated an SP triboelectric sensor for monitoring dopamine in the alkaline condition on the bases of TENG, which was composed of PTFE with nanoparticle arrays and an Al film (Figures 4(c) and 4(d)) [94]. Because the nano-stick PTFE exhibits a strong interaction of dopamine, the output performance of the TENG was inversely proportional to the concentration of dopamine in the range of 10 *μ*M-1 000 *μ*M (Figure 4(e)). In this work, a detection limit of dopamine was 0.5 *μ*M, which suggested an effective means of SP electrochemical sensor for dopamine detection. Wen et al. introduced an SP gas sensor on the basis of a blow-driven TENG, where a rotary TENG was applied as shown in Figure 4(f) [95]. The illustration of the SP breath analyzer is shown in Figure 4(g). Driven by mouth blowing, the output voltage of the sensor was only proportional to the concentration of alcohol in the airflow (Figure 4(h)). On the basis of blow-driven TENG, the active alcohol breath analyzer exhibited a high detection gas response of ~34 under an optimized condition. Additionally, when the blow-driven TENG was blew by a tester without drinking alcohol, the voltage drop across the sensor was almost zero due to a low sensor resistance (Figure 4(i)). While the air-flow of a tester that had been drank across the sensor, an enhanced voltage would be generated and thus could trigger the warning system (Figure 4(j)), which was caused by the dramatically increased resistance of the sensor in the breathed-out alcohol vapor.

### 3.4. Self-Powered Electrochromic System

Electrochromic devices can reversibly change their optical properties by the electrochemical redox reaction under an external electric field [96]. Applied TENG as the electricity source to provide a constant voltage, SP electrochromic device has been realized to replace batteries, which provides a promising sustainable power solution [97, 98].

**Figure 5:**
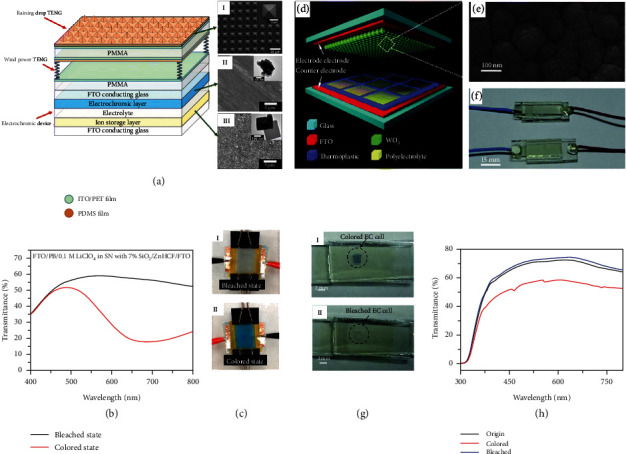
Self-powered electrochromic reaction: (a) schematic diagram of the SP smart window integrated with a raindrop-TENG, a wind-powered-TENG, and an electrochromic device from the top to the bottom; inserts are the SEM images of (I) PDMS film, (II) PB film, and the (III) ZnHCF film; (b) transmittance spectra and (c) photographs of the SP smart window system in bleached and colored states [99]; (d) the schematic structure of the WO_3_-based SP electrochromic device; (e) SEM image of the WO_3_ film; (f) the photographs of the EC WO_3_-based SP electrochromic device; (g) color changing images of the WO_3_-based SP electrochromic device; (h) transmittance spectra of the WO_3_-based SP electrochromic device [100].

Driven by a dual-mode TENG for harvesting wind and raindrop energy, Yeh et al. realized an SP smart window system [99]. The dual-mode TENG involved a single-electrode TENG on the top of the SP smart window for harvesting the energy from raindrop motions, and a contact-mode TENG assembled by elastic springs below the abovementioned single-electrode-mode TENG for collecting energy from wind energy. Both of the two TENGs consisted of a PDMS thin film adhered to a conducting substrate that were adhered to the electrochromic device with a substrate to fabricate an SP system (Figure 5(a)). The electrochromic device mainly consisted of Prussian blue (PB) nanoparticles and zinc hexacyanoferrate (ZnHCF) nanocubes as the electrochromic material and the ion storage layer. By photolithography and a template molding process, the PDMS film has a micropatterned pyramid array structure for enhancing the hydrophobic property and effect contact area of the surface. Driven by the dual-TENG, the transmittance of the electrochromic exhibited reversible variations. For gaining a more intuitive view of the change of the optical property, the transmittance of the electrochromic device was measured from 400 to 800 nm both before and after the coloring process. As shown in Figure 5(b), the transmittance declined in the full range during the coloring process, and the highest variation was realized at 695 nm, while the transmittance declined from 53.5% to 20.9%. These transmittance changes can be observed by visualization, where the color of the electrochromic device changed from transparent in bleached state to deep blue in colored state (Figure 5(c)). In this work, 32.4% of the maximum transmittance change was achieved at 695 nm, which closed to the value of 32.6% that powered by a conventional electrochemical power source.

Yang et al. reported a WO_3_-based electrochromic device integrating with TENG to fabricate an SP electrochromic device [100]. The SP electrochromic device had a multilayered structure, which is displayed in Figure 5(d). Commercial glass was used as the substrate, on which there was a layer of FTO thin films acted as electrodes. The sheet resistance and transmittance of FTO film were 35-45 *Ω* per sq. and 80%, respectively. Between the two electrodes, there were an array of cells and a layer of WO_3_ film (Figure 5(e)), and the distance between the two electrodes was around 20 mm. The cells were filled with polyelectrolyte, and the WO_3_ film was about 250 nm which was densely packed nanoparticles. As shown in Figure 5(f), the fully packaged SP electrochromic device still had a transmittance of more than 70%. As shown in Figure 5(g), powered by TENG, the transmittance of electrochromic cell was drop via coloring process (Figure 5(g), (I)). When the connection of reversible switch was reversed, the electrochromic cell returned to transparency (Figure 5(g), (II)). Compared with the SP electrochromic device, the transmittance of the device greatly dropped, and a relatively stable difference of as much as 17% was maintained for wavelength that ranges from 450 nm to 650 nm (Figure 5(h)), indicating the sensitivity and the applicable to a broad range of light wavelength.

To effectively harvest acoustic energy, Qiu et al. integrated a sandwich-like structured TENG with electrochromic device for reversible color changing [101]. The TENG consisted of three layers including Cu foam, polyvinylidene fluoride (PVDF) nanofiber, and a nylon fabric. There was a thick spacer layer between the nylon fabric and PVDF nanofibers to produce more space vibration for the membrane. Driven by sound, high outputs of 25.01 mA m^−2^ and 20.91 *μ*C s^−1^ were achieved for the designed TENG. The high performance of TENG enabled it to power an electrochromic device. In this system, reversible switches controlled the oxidation and reduction process and thus controlled the color change. Driven by the TENG, the color of electrochromic film changed between the transparent white and dark blue, which were controlled by the switching dote.

### 3.5. Self-Powered Anticorrosion System

Material corrosion is a long-standing challenge in many engineering applications [102]. The cathodic protection is one of the most durable methods to protect the materials from corrosion, which involves sacrificial anode cathodic protection system (SACPS) and impressed current cathodic protection system (ICCPS) [103–105]. Different from SACPS, the ICCPS can protect the steel by cathodic current from a direct current source without any sacrifice of electrode materials [106]. However, the requirement of external electric energy supply limits its practical application. For solving this issue, many works have been focused on the SP anticorrosion system by integrating TENG with chemical anticorrosion protection [107–111].

**Figure 6:**
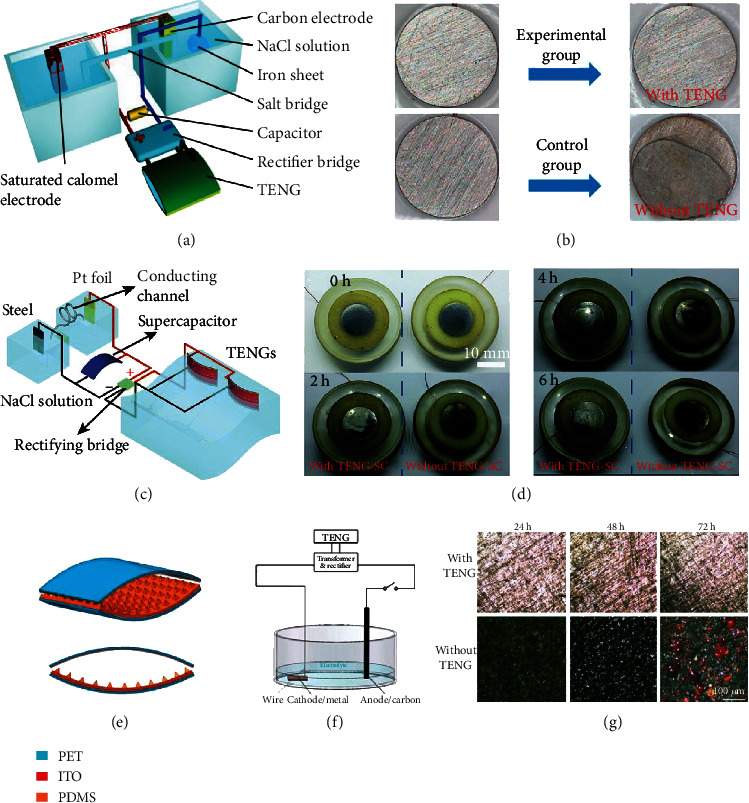
Self-powered anticorrosion system: (a) sketch map of the SP anticorrosion system; (b) iron sheets before and after soaked in the solution for 2 h [112]; (c) sketch map of the SP anticorrosion system powered by TENG; (d) the contrast images of the surface morphology of the samples after immersing in sodium chloride solution driven by TENG or not [113]; (e) scheme of the designed TENG; (f) schematic diagram of the metal surface cathodic protection system driven by TENG; (g) micrographs of the rusted specimens after accelerated corrosion [110].

Wang et al. realized an SP anticorrosion system for iron sheet driven by a high performance TENG [112]. Utilizing the fabricated nanostructures and prior-charge injection method, the charge densities were improved by 48% and 53%, respectively. The schematic image of SP anticorrosion system is depicted in Figure 6(a). Aiming to study the effect of SP anticorrosion system, the ion steel was soaked into 3.5% NaCl solution for 2 h, and the corresponding surface morphology is displayed in Figure 6(b). As displayed, almost no change of the iron sheet was observed by using TENG, while a thin rust film was produced without TENG. This result indicated a good anticorrosion property of the SP system, which exhibited a good prospect to protect materials from rusting with low energy cost.

Considering the metal corrosion is more likely to happen under ocean environmental conditions, Li et al. developed an SP system on the basis of networking TENG and supercapacitor to convert water wave to electricity for metal anticorrosion [113]. To obtain a stable and continuous output, the flexible TENG integrated with a flexible double-layer supercapacitor for harvesting the wave energy and then storing the energy in the supercapacitor. The structure and working mechanism of the SP system displayed in Figure 6(c). Driven by the water wave in the condition of 0.2 m s^−1^ and 1 Hz, the protected steels injected into electrons stemmed from TENG, leading to a cathodic polarization. In this work, a declined potential of steel electrode from -0.35 V to -0.6 V indicated that the steel become more stable in the protection of designed SP system. Immersing in the solution of sodium chloride with concentration of 0.5 M, there was a thin layer of rust on the steel without the protection of SP system, while there were a few corrosion pits on the steel when the SP system was working (Figure 6(d)). These results demonstrated that the SP system significantly decreased the corrosion rate, which could be adopted in marine corrosion.

Zhu et al. designed an SP cathodic protection system based on a flexible TENG, which can harvest energy from natural rain drops and wind to drive the cathodic protection process [110]. The contact-separation-type TENG was mainly composed of PDMS film and ITO, which were acted as a pair of triboelectric layers (Figure 6(e)). The schematic diagram of the SP metal surface cathodic protection system is shown in Figure 6(f). Under stimulations at a frequency of 1 Hz, the output current of the TENG was over 130 *μ*A, and the voltage reached about 500 V. In this SP cathodic protection system, the protected metal that was immersed into the electrolyte was acted as the cathode, while a carbon rod was used as the anode. The comparison results of the rested specimens with and without SP cathodic protection system are displayed in Figure 6(g). For the specimens protected with SP cathodic protection system, several gray distributing areas appeared on the surface of specimens. These areas enlarge with the increase of corrosion, but no apparent corrosion was observed on these samples. As for the specimen without SP cathodic protection system powered by TENG, numerous corrosion pits were found on the three surfaces of specimens, and more big pits arose on the sample that was corroded in simulated electrolyte for 72 h.

## 4. Summary and Perspective

Electrochemistry has brought earth-shaking changes to our life, and it has become a technology for production and research in many fields. In order to break the limitation of external powers source for electrochemical operation, integrating electrochemical system with TENG to form SPES is the most promising strategies. According to the latest achievements, SPESs can be summarized into five major fields including pollutants treatment, electrochemical synthesis, electrochemical sensor, electrochromic reaction, and metal anticorrosion, respectively. Although researches into SPES have realized remarkable progresses, the following issues should be addressed for promoting further development of this field:

1. *High Output Power and Durability of TENG*. Output power and durability of TENG are two key points to realize high performance of SPES. To improve the output power of TENG, further improving the surface charge density and integrating existing methods in a large scale are suggested. To enhance the durability of TENG, introducing interface liquid lubrication [114] and developing materials with the most robust mechanical durability and stability might be the promising strategies
2. *Power Circuit Management of TENG*. TENG has the characteristics of high voltage output and low current output, while the performance of electrochemical process exhibits positive correlation with current density and negative influence such as passive electrode effect and secondary reaction induced by high potential. To achieve a high performance of SPES, the power circuit management of TENG is highly desired to match the corresponding electrochemical process
3. Reducing electrode passive is crucial to prolong the lifetime of electrodes, improve the electrochemical efficiency, and ensure a stable activity. TENG with a pulse output signal has been reported to reduce the electrode passive effect; however, the phase superposition of TENG caused by multiple parallel electrodes makes it hard to realize a full-waveform-pulse-current. Therefore, rational design of the structure of TENG, such as adjusting the rotation center angle ratio between each rotator and stator to obtain a full-waveform-pulse-current, will be a promising strategy to optimize the processes of SPES. It has been reported that alternating current exhibits many virtues such as lower energy consumption, improved mass transfer characteristic, and delayed electrode passivation compared to direct current in the electrochemical system [115]; thus, utilizing the alternating current of TENG to build SP electrode system is the other method to reduce the electrode passive and improve the electrochemical performance
4. The electrode plays a key role in the electrochemical reaction. Therefore, the electrode materials are the most key factors to determine the properties of electrochemical reaction. Novel electrode materials such as nanoscale electrode materials and metal-organic framework materials with merits of high conductivity, high specific surface areas, high activity, and cycle stability should be prepared to further enhance the performance of SPES
5. *New Application of SPES*. With the improvement performance of TENG, it can be utilized as electricity source to power some new electrochemical reaction such as electrocoagulation for removal of oil in water, electrodialysis for desalination and water reuse, and electrophoresis for the separation of protein to build SPES for overcoming the problem of external power supply. Besides traditional electrochemistry based on electrolytic cell that needs high current density, the interrelation of electrical and chemical effect can also be realized by high voltage electrostatic discharge. By utilizing TENG, a high voltage electrostatic is actually quite easy to achieve. Therefore, integrating TENG with electrochemical system to build SP discharge electrochemical system for removing pollutants such as PM [63] provides a new horizon for its application
